# Anticoagulation Therapy for Pregnancy-Associated Thrombosis: A Retrospective Observational Study

**DOI:** 10.3400/avd.oa.22-00083

**Published:** 2022-12-25

**Authors:** Michihisa Umetsu, Daijirou Akamatsu, Fukashi Serizawa, Yuta Tajima, Shunya Suzuki, Shinichiro Horii, Norinobu Ogasawara, Hirokazu Takahashi, Yohei Nagaoka, Kota Shimizu, Shunsaku Kimura, Munetaka Hashimoto, Hitoshi Goto, Tetsuo Watanabe, Takashi Kamei

**Affiliations:** 1 Tohoku University Hospital, Sendai, Miyagi, Japan; 2 Iwate Prefectural Isawa Hospital, Ohshu, Iwate, Japan; 3 South Miyagi Medical Center, Shibata, Miyagi, Japan; 4 Sendai City Hospital, Sendai, Miyagi, Japan

**Keywords:** pregnancy, anticoagulation, DVT, VTE, heparin

## Abstract

**Objectives:** Pregnancy-associated deep vein thrombosis (DVT) is a rare disease, and data on anticoagulation therapy are lacking. The present study examined the treatment outcome with unfractionated heparin (UFH) subcutaneous injection in patients with pregnancy-associated DVT.

**Methods:** This single-center, retrospective, observational study enrolled 15 patients with pregnancy-associated DVT treated from January 2014 to April 2021.

**Results:** The median age was 35 years. The median gestation week at onset was 10 (interquartile range is 8–11). All patients presented with painful symptoms with edema. All patients had proximal DVT. Anticoagulation therapy using UFH was performed in 14 patients. The median continuous dose of heparin was 18,750 U/day, and the median subcutaneous dose was 20,000 U/day. During the outpatient period, the values of activated partial thromboplastin time fluctuated wildly, but the fibrin monomer complex level remained consistently low. There were two mild bleeding complications, but neither prevented the continuation of anticoagulation therapy. During delivery, thrombi were not detected in 10 of 13 patients (77%), whereas three patients (23%) exhibited regression without resolution of the thrombus.

**Conclusion:** Anticoagulation using UFH subcutaneous injection was safely performed in patients with pregnancy-associated DVT without serious complications or progression of thrombosis.

## Introduction

Pregnancy is a condition associated with multiple risk factors for venous thromboembolism (VTE), including elevated levels of various coagulation factors, low protein S levels, and dehydration attributable to malabsorption. The elevated coagulation factors include factors X, IX, V, and VIII, which play an essential role in thrombin generation, and factor VII, which initiates blood coagulation with tissue factor. In addition, decreased protein S levels tend to increase the risk of thrombus formation.^1)^ In addition, some patients have undetected hereditary thrombophilia. The incidence of pregnancy-associated deep vein thrombosis (DVT) in western countries has been reported to range from 7.2–12 per 10,000 deliveries.^2,3)^ The incidence of the disease in Japan is 7.5 per 10,000 pregnant women, according to a nationwide epidemiological survey conducted by the Japan Environment and Children’s Study Group.^4)^

Meanwhile, pregnancy-associated thromboembolism increases Japan’s risk of maternal death.^5)^ The available anticoagulants for pregnant women with DVT are limited because of teratogenicity and placental transfer risks. The American Society of Hematology clinical guidelines recommend low-molecular-weight heparin (LMWH) for pregnant women with acute VTE. However, LMWH is not permitted for VTE treatment in Japan. Contrarily, Japanese guidelines recommend using unfractionated heparin (UFH) for antithrombotic prophylaxis in high-risk pregnancies.^6,7)^

There are racial differences in the propensity for thrombus formation. Hereditary thrombophilia, such as that associated factor V Leiden and the prothrombin G20210A gene variant, has been reported in Caucasians, but such findings have not been reported in Asians.^8)^ Conversely, protein S deficiency is more common in Japan than in other East Asian countries because of the protein S K196E mutation.^9,10)^ In Japan, several case reports on DVT associated with pregnancy have been published, but there have been no comprehensive studies. Therefore, we conducted a retrospective, observational study of pregnancy-associated DVT in our institute.

## Materials and Methods

### Ethics approval and consent to participate

All procedures were per the Declaration of Helsinki. The Institutional Review Board approved this study of Tohoku University Graduate School of Medicine (2019-1-764). The requirement for written informed consent was waived because the clinical information was obtained in routine clinical practice. This method concurred with the Guidelines for Epidemiological Studies issued by Japan’s Ministry of Health, Labour, and Welfare.

### Study population

**Figure 1:**
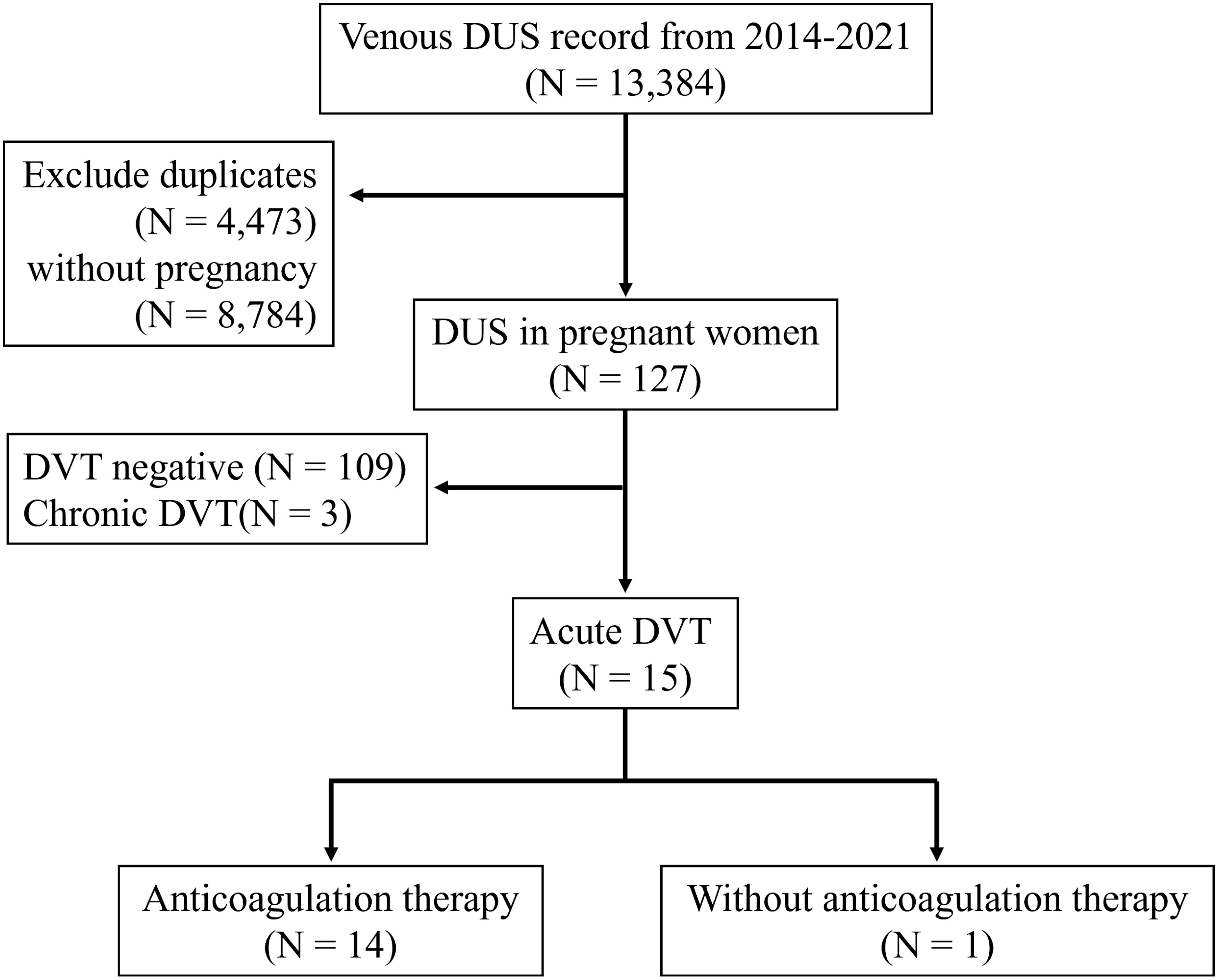
Fig. 1 Flowchart for patient selection.

DUS: duplex ultrasound scanning; DVT: deep vein thrombosis

Whole-leg compression duplex ultrasound (DUS) was performed on 13,384 patients from January 2014 to April 2021 at our physiological laboratory center. Among these, 4,473 duplicated patients were excluded. Meanwhile, 127 pregnant women with suspected pregnancy-associated DVT were eligible for enrollment. Of these, 18 patients were diagnosed with DVT, and patients with chronic DVT and no change in location were excluded. Finally, 15 patients with acute pregnancy-associated DVT were enrolled. **Figure 1** presents the flow diagram for patient selection.

### Data collection

Data on the patient’s basal characteristics, the physical examination findings on arrival, the treatment management, the outpatient treatment course, complications of bleeding or pregnancy, and treatment for the subsequent pregnancy were retrospectively collected from medical records.

### Treatment procedure for pregnancy-associated DVT

The general treatment of patients with pregnancy-associated DVT at our institute was as follows. When DVT was suspected, patients were referred from neighboring facilities to the Department of Obstetrics of our institution. If proximal DVT was present after blood sampling and lower-extremity venous DUS, the patient was admitted to the Department of Obstetrics. The Department of Obstetrics provided maternal and fetal care, and vascular surgeons provided DVT treatment. First, UFH was administered continuously. When the activated partial thromboplastin time (APTT) was prolonged and the fibrin monomer complex (FMC) level was decreased, the patient was switched to heparin calcium subcutaneous injection. The subcutaneous injection was divided into two daily doses administered at 8:00 and 20:00. Because heparin calcium was a 5,000 U/0.2 mL syringe formulation, if there was an excess portion, the same amount was used according to the proficiency of the technique, or if it was difficult for the patient to receive, the injection time was changed according to the amount of UFH (e.g., 25,000 U/day; 12,500 U twice daily at 8:00 and 20:00; 10,000 U at 11:00 and 15,000 U at 20:00). If the patient successfully learned the subcutaneous injection technique, she was discharged for outpatient treatment. The patient was visited every 2–4 weeks during outpatient treatment. Venous DUS was performed at the physiological laboratory center approximately every 3 months. The delivery method was not restricted and was selected at the discretion of the obstetricians.

### Definitions for patient characteristics

Proximal DVT was defined as thrombosis in central veins, including the popliteal, femoral, and iliac veins and the inferior vena cava. Submassive pulmonary thromboembolism (PTE) was defined as acute PTE without systemic hypotension but with right ventricular dysfunction.

### Statistical analysis

Categorical data were expressed as numbers and percentages, and continuous data were expressed as the median and interquartile range (IQR). All statistical analyses were performed using JMP Pro version 15.0.0 software (SAS Institute Inc., Cary, NC, USA).

## Results

### Patient characteristics

Between January 2014 and April 2021, we identified 15 patients with pregnancy-associated DVT. The patient characteristics are presented in **Table 1**. The median patient age was 35 years (range 26–40), and the median body mass index was 21.7 kg/m^2^ (IQR=20.6–23.8). The previous gravidity was observed in five patients (33%), previous parity was observed in three patients (20%), and spontaneous abortion was observed in three patients (20%). The median gestation week at onset was 10 (IQR=8–11). Lower-extremity edema was the main complaint in all patients, and it was associated with leg pain in 14 patients (93%), and leg cramps in one patient (7%). All patients presented with painful symptoms as well as edema. The comorbidities were antiphospholipid syndrome in one patient and previous DVT in three patients.

**Table 1:** Table 1 Patient characteristics and thrombus information

	PA-DVT (N=15)
Age (years, median)	35 (range, 26–40)
BMI (kg/m^2^, median)	21.7 (IQR=20.6–23.8)
Previous gravidity (N, %)	5 (33%)
Previous parity (N, %)	3 (20%)
Previous spontaneous abortion (N, %)	3 (20%)
Weeks of gestation at onset (weeks, median)	10 (IQR=8–11)
Complaints (N, %)	
Edema	15 (100%)
Pain	14 (93%)
Leg cramps	1 (7%)
Comorbidities (N, %)	
APS	1 (7%)
Protein C deficiency	1 (7%)
Protein S deficiency	1 (7%)
Previous DVT	3 (20%)
DVT location	
Right/left (N, %)	5 (33%)/10 (67%)
Proximal/distal (N, %)	15 (100%)/0 (0%)
Most proximal region of thrombus	
Iliac vein (N, %)	9 (60%)
Femoral vein (N, %)	3 (20%)
Popliteal vein (N, %)	3 (20%)
Symptomatic PTE (N, %)	1 (7%)
D-dimer (µg/mL, median)	9.3 (IQR=3.8–13.4)
FMC (µg/mL, median)	45 (IQR=4.8–150*)
Protein S (%, N, %)	37 (IQR=26–85), low 9 (60%)
Protein C (%, N, %)	100 (IQR=82–115), low 2 (13%)
Antithrombin (%)	95 (IQR=81–98)
LAC-positive (N, %)	1 (7%)

PA-DVT: pregnancy-associated deep vein thrombosis; BMI: body mass index; IQR: interquartile range; APS: antiphospholipid antibody syndrome; DVT: deep vein thrombosis; PTE: pulmonary thromboembolism; FMC: fibrin monomer complex; LAC: lupus anticoagulant *Measurement limit value

### Thrombus information

All patients had proximal DVT; DVT was right-sided in five patients (33%) and left-sided in 10 patients (67%). The most proximal region of DVT was the iliac, femoral, and popliteal veins in nine (60%), three (20%), and three patients (20%), respectively. The locations of DVT in all 15 cases are shown as follows: iliac to infrapopliteal vein, 6; iliac to femoral vein, 2; iliac vein only, 1; femoral to infrapopliteal vein, 3; and popliteal to infrapopliteal vein, 3. One patient had submassive PTE. The median D-dimer level was 9.3 µg/mL (IQR=3.8–13.4), and the median FMC level was 45 µg/mL (IQR 4.8–over 150) at the first arrival. The median protein S value was 37% (IQR=26–85), and the value was low in nine patients (60%). The median protein C level was 100% (IQR 82–115), and the level was low in two patients (13%). The median antithrombin level was 95% (IQR=81–98), and the lupus anticoagulant was detected in one patient.

### Anticoagulation treatment

Fourteen patients received anticoagulation using UFH upon hospitalization. In addition, one patient with a previous episode of proximal DVT had a minor recurrence, and she was treated as an outpatient with compression stockings. **Table 2** presents the treatment procedures during hospitalization. The median continuous dose of heparin was 18,750 U/day (IQR=15,000–25,750). The median subcutaneous injection dose was 20,000 U/day (IQR=15,000–25,000). The median time to transition from continuous to subcutaneous injection was 10 days, and the median hospital stay was 19 days (IQR=14–26). Compression stockings were used as adjunctive therapy in all patients, and no patients were treated with inferior vena cava filters.

**Table 2:** Table 2 Treatment during hospitalization

	PA-DVT (N=15)
Unfractionated heparin	
Continuous dose of heparin (U/day)	18,750 (IQR=15,000–25,750)
Subcutaneous injection dose (U/day)	20,000 (IQR=15,000–25,000)
Continuous to subcutaneous injection (days)	10 (IQR=9–13)
Hospital stay (days)	19 (IQR=14–26)
Adjunctive therapy	
Compression stockings (N, %)	15 (100%)
IVC filter (N, %)	0 (0%)

PA-DVT: pregnancy-associated deep vein thrombosis; IQR: interquartile range; IVC: inferior vena cava

### Outpatient management

**Figure 2:**
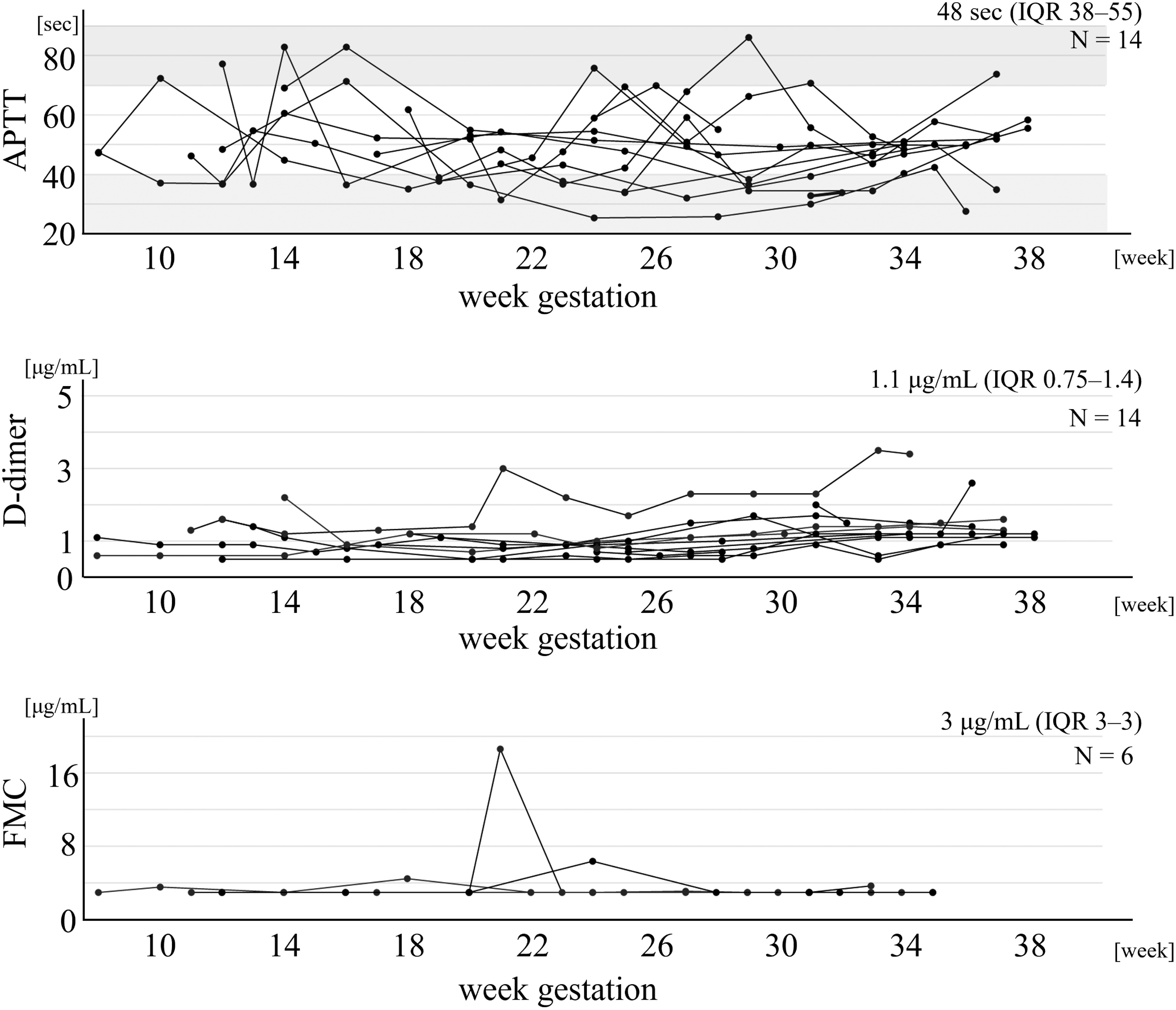
Fig. 2 Trends in APTT, D-dimer, and FMC during outpatient treatment. APTT, D-dimer, and FMC are presented on the vertical axis, and the weeks of gestation are presented on the horizontal axis. The APTT fluctuated wildly. D-dimer levels were generally low during pregnancy but tended to increase after 30 weeks of gestation. FMC levels remained consistently low throughout pregnancy.

APTT: activated partial thromboplastin time; FMC: fibrin monomer complex

Blood coagulation parameters during outpatient subcutaneous injection therapy are presented in **Fig. 2**. The median APTT was 48 s (IQR 38–55), but the values fluctuated wildly. D-dimer levels were constantly low, with a median level of 1.1 µg/mL (IQR=0.75–1.4), but there was an upward trend after 30 weeks of gestation. FMC levels remained consistently low regardless of the gestational time. FMC levels were temporarily elevated in one patient, but this coincided with the onset of mild placental bleeding. The APTT was prolonged to more than 70 s on eight occasions in six patients. The injection time and blood collection time were apparent in four patients, and all four underwent blood collection tests within 2–2.5 h after subcutaneous injection. Three patients continued on the same dose of heparin, and their APTTs were within the target range at the next arrival. The remaining patient received a reduced dose of heparin, and her APTT was within the target range at the next arrival.

### Complications

No patients experienced skin complications attributable to compression stockings; however, redness and pruritus were observed in three patients (20%) because of subcutaneous injection. An ointment improved all symptoms, and subcutaneous heparin treatment could be continued. Bleeding complications were observed in two patients (14%), including one case each of genital and placental bleeding. Both complications were mild, and subcutaneous heparin could be continued. One patient experienced an exacerbation of systemic lupus erythematosus as a pregnancy complication that resulted in an emergency cesarean section. Gestational hypertension was observed in another patient.

### Delivery and the subsequent outcome

Vaginal delivery was selected in 10 patients (67%), and cesarean section was selected in five patients (33%). Heparin was terminated 6 h before cesarean section or planned delivery.

After discharge, patients received anticoagulation with warfarin if suspected of hereditary thrombophilia. The anticoagulation was judged individually according to the bleeding risk and thrombus regression when the presence of thrombophilia was unclear. After delivery, warfarin therapy was started in seven patients (47%), and eight patients (53%) were observed without anticoagulation.

Eleven patients were assessed for subsequent pregnancies. Five patients had a subsequent pregnancy and delivery, and all five received subcutaneous heparin calcium. The dose of subcutaneous injection was based on the previous dose, and no inpatient treatment was given. Meanwhile, one patient had a miscarriage, and the remaining had no pregnancies.

Among the 14 patients who underwent venous DUS until the remote postpartum period, two were thrombus-free, and 12 had a regression of the thrombus. Eight of these 12 patients were nearly thrombus-free, with only linear fibrin structures. Two patients had residual occlusion of the femoral vein. In one case, the femoral vein was already occluded by previous DVT. The other case was an occlusive DVT with abundant thrombus from the iliac vein to the infrapopliteal vein, which the femoral vein diameter was decreased and occluded. Initial symptoms were improved in all cases. In the late phase, there were cases with residual symptoms of 1–2 points on the Villalta scale, such as a left-right difference in circumference and a left-right difference in tiredness, no moderate or severe post-thrombotic syndrome was observed.

## Discussion

In the present study, anticoagulation with subcutaneous heparin calcium was safely performed in patients with pregnancy-associated DVT. All patients were delivered safely with no serious complications or progression of thrombosis. Subcutaneous injection of heparin calcium was associated with several features, such as temporary hospitalization to learn self-injection techniques, inconsistency of the APTT in the outpatient period, and a lack of awareness of mild skin complications at the subcutaneous injection site. The combined D-dimer and FMC measurements in the outpatient period appeared to reduce excessive prolongation and safe treatment.

Although there have been reports of LMWH treatment for pregnancy-associated DVT, no recent studies have discussed using UFH for this indication. Because some patients cannot receive LMWH, such as those with renal dysfunction, and because LMWH is not permitted for DVT treatment in some countries, we believe that UFH treatment remains a currently used therapy. In addition, no reports provided detailed descriptions of clinical techniques and precautions for subcutaneous injection therapy. This study is novel because it describes clinically relevant issues in detail.

The factors that prevented stabilization of the APTT in the therapeutic range included the timing of subcutaneous injection and blood sampling. According to the drug information, the plasma levels of heparin calcium peak 2–3 h after subcutaneous administration and then decline. When the APTT is prolonged, the time of subcutaneous injection and blood collection must be determined before reducing the heparin dose. To prevent an excessive prolongation of the APTT, it may make sense to perform the measurement at a time closer to the peak and to maintain the therapeutic dose for a longer period. It may be better to use the trough level. VTE guidelines advocate for blood sampling at a point between injections.^7)^ However, in the real world, adjusting the timing of injection and blood sampling is difficult, depending on various factors. The timing of subcutaneous injection was standardized to 8:00 am and 8:00 pm at the time of hospitalization. However, at the time of discharge, the timing varied depending on each patient’s lifestyle.

D-dimer and FMC are valuable indicators of the absence of new thrombotic tendencies. D-dimer is a general term for the degradation products of stabilized fibrin. It is helpful as an indicator of the presence of a thrombus because its levels increase with secondary fibrinolysis. However, D-dimer is not a highly specific marker, as its levels tend to increase in the third trimester. Conversely, FMC is a more sensitive marker of thrombosis. In this study, FMC levels were low, and no thrombotic tendency was observed. Its levels increased only during placental bleeding, which may reflect thrombus formation in the placenta. Therefore, the APTT should be used to confirm the absence of excessive prolongation of heparin, and D-dimer and FMC should be used as indicators of the presence or absence of DVT exacerbation.

When subcutaneous injection was performed, skin pigmentation and induration were observed. In some cases, when the subcutaneous injection was performed on the indurated area, heparin calcium did not penetrate sufficiently into the skin, and it leaked from the body. Although we are instructed to rotate the site of subcutaneous injection to the thighs and abdomen, it is necessary to notify the patient to shift the injection by several centimeters in the same area and avoid induration. When the amount of heparin was 20,000 U/day, it should be injected at a volume of 0.4 mL at a time. It should be recognized that this volume is larger than that of a typical insulin preparation (100 U/mL).

A serious side effect of heparin is heparin-induced thrombocytopenia (HIT), which is a prothrombotic adverse reaction that occurs in <0.1–7% of patients treated with heparin products.^11)^ In this study, no cases of thrombocytopenia would raise suspicion of HIT. A report stated thromboprophylaxis with LMWH during pregnancy did not cause HIT.^12)^ In particular, UFH carries a higher HIT risk than LMWH and requires more attention.^13)^ Furthermore, UFH carries a risk of osteoporotic fractures. Osteoporotic vertebral fractures were found in 2.2% of pregnant women who used heparin.^14)^ In this study, no patient complained of osteoporotic fracture or back pain. We must treat late phase osteoporosis when using large amounts of UFH. Thus, measurements of D-dimer and FMC levels could help prevent excessive increases in the heparin dose that could lead to preventing HIT and osteoporosis.

Anticoagulation therapy has greatly progressed over the past quarter-century. Various coagulation factor inhibitors have been invented, and in recent years, the usefulness of direct oral anticoagulants (DOACs) compared to warfarin or LMWH has been reported in patients with malignancies.^15,16)^ During pregnancy, the risk of bleeding and placental transfer must be considered. Heparin is considered the safest drug because it does not cross the placenta, and in some countries, subcutaneous LMWH injection has been used for decades.^17)^ Warfarin crosses the placenta and carries a 30% risk of causing nasal dysplasia, epiphyseal dysplasia, and neurodevelopmental abnormalities during the organogenesis period at 6–12 weeks gestation. Therefore, this treatment is not recommended unless the benefits outweigh the risks, such as after valve replacement surgery.^18)^ DOACs have been clinically applied for VTE since 2005 in Japan, but they are placenta-transferable. The accidental use of DOACs during pregnancy has been described, and fetal and neonatal abnormalities were observed in children born to mothers who received rivaroxaban.^17)^ Concerning other DOACs, although case reports indicated that they could be used without major complications, there is no accumulated evidence confirming their safety. With the accumulation of further evidence, a simpler alternative to subcutaneous injection is possible.

Study limitations: This study had several limitations. One limitation was that this was a single-center, single-arm, retrospective, observational study. In addition, the sample size was small. Meanwhile, it was not easy to match women by age comparably; thus, a two-arm comparison study was unfeasible. Because pregnancy-associated DVT is rare, it is difficult to accumulate prospective cases in a single-center study, and a multicenter study is desirable. In addition, distal isolated DVT could be neglected because DVT screening DUS was not performed for all the pregnant women.

## Conclusion

Anticoagulation using subcutaneous UFH injection was safely performed for pregnancy-associated DVT with no serious complications or progression of thrombosis.
